# Rhein prevents endotoxin-induced acute kidney injury by inhibiting NF-κB activities

**DOI:** 10.1038/srep11822

**Published:** 2015-07-07

**Authors:** Chen Yu, Dong Qi, Ju-Feng Sun, Peng Li, Hua-Ying Fan

**Affiliations:** 1 School of Pharmacy, Binzhou Medical University, Yantai, Shandong, China; 2 Department of Nephrology, Yu-Huang-Ding Hospital/Qingdao University, Yantai, Shandong, China; 3 School of Pharmacy, Yantai University, Yantai, Shandong, China

## Abstract

This study aimed to explore the effect and mechanisms of rhein on sepsis-induced acute kidney injury by injecting lipopolysaccharide (LPS) and cecal ligation and puncture (CLP) *in vivo*, and on LPS-induced HK-2 cells *in vitro*. For histopathological analysis, rhein effectively attenuated the severity of renal injury. Rhein could significantly decrease concentration of BUN and SCr and level of TNF-α and IL-1β in two different mouse models of experimental sepsis. Moreover, rhein could markedly attenuate circulating leukocyte infiltration and enhance phagocytic activity of macrophages partly impaired at 12 h after CLP. Rhein could enhance cell viability and suppresse the release of MCP-1 and IL-8 in LPS-stimulated HK-2 cells Furthermore, rhein down regulated the expression of phosphorylated NF-κB p65, IκBα and IKKβ stimulated by LPS both *in vivo* and *in vitro*. All these results suggest that rhein has protective effects on endotoxin-induced kidney injury. The underlying mechanism of rhein on anti-endotoxin kidney injury may be closely related with its anti-inflammatory and immunomodulatory properties by decreasing NF-κB activation through restraining the expression and phosphorylation of the relevant proteins in NF-κB signal pathway, hindering transcription of NF-κB p65.These evidence suggest that rhein has a potential application to treat endotoxemia-associated acute kidney injury.

Acute kidney injury (AKI) is common organ injury in intensive care unit (ICU) and is associated with higher resource utilization and mortality than that of other critical care syndromes^1^. Currently, there is no effective treatment except renal replacement therapy (RRT)^2^,^3^,^4^. AKI is a frequent and serious complication of sepsis in ICU patients^5^. Sepsis and its most severe presentation, septic shock, are the main causes of AKI in the ICU; accounting for up to 50% of all cases^6^.Sepsis-induced AKI is associated with high mortality^7^. The overall mortality rate of AKI is about 45%; however, the mortality rate of sepsis-induced AKI is about 70%^8^.

Although sepsis is the most common trigger of AKI, the underlying mechanisms are not completely known. More and more studies have revealed that the pathophysiology of AKI in sepsis is complex and multi-factorial, including inflammatory pathways, intrarenal hemodynamic changes, endothelial dysfunction, microcirculatory disorders, intraglomerular thrombosis and so on^9^. The inflammatory response inherent to sepsis has been considered as a direct mechanism of AKI^10^. A growing body of evidence suggests that AKI in sepsis has a prominent inflammatory component both in the initiation and extension phase of the kidney injury^11^. Previous studies demonstrated that during sepsis, repeated stimulation of the kidneys led to the production of a large number of pro-inflammatory cytokines including tumor necrosis factor (TNF) -α, interleukin (IL) -6 through nuclear factor-κappa B (NF-κB) signaling pathway, which is a key regulator in keeping homeostasis of immune system and a major drug target in a variety of diseases^12^,^13^,^14^.

Rhubarb (*Rheum officinale*, Polygonaceae), which is one of popular traditional Chinese herbal medicine, has been widely used for the treatment of renal diseases and infectious inflammation in traditional Chinese medicine for many years^15^. Rhein (4, 5- dihydroxyanthraquinone-2-carboxylic acid, its chemical structure is shown in Fig. 1) is one of the most important bioactive anthraquinone derivatives from rhubarb. Recent studies have shown that rhein could inhibit NF-κB activation and sequentially suppresses its downstream inflammatory cytokines transcriptions in lipopolysaccharide (LPS)-activated macrophages^16^. Numerous earlier reports have identified that rhein can ameliorate glomerular hypertrophy, reduce urinary protein, suppress renal fibrosis in diabetic nephropathy, and improve renal function, reduce renal cell apoptosis to protect against further progression of chronic renal failure^17^,^18^,^19^,^20^,^21^.

However, the effects and mechanisms of rhein on AKI are still unclear. In this study, we designed and performed experiments to investigate the effect and mechanism of rhein on sepsis-induced AKI in two different *in vivo* and *in vitro* models. This study presented new insights into mechanisms of rhein against AKI and provided pharmacological evidence for clinical applications.

## Materials and Methods

### Chemicals and reagents

Rhein were purchased from National Institute for the Control of Pharmaceutical and Biological Products (Beijing, China). The purity of the compound rhein was certified to be 98% by high-performance liquid chromatography (HPLC). Lipopolysaccharide (LPS) from Escherichia coli 055:B5 was obtained from Sigma-Aldrich Chemical Co. (USA). TNF-α, IL-1β, monocyte chemoattractant protein (MCP)-1 and IL-8 ELISA Kits were purchased from Yantai Science & Biotechnology (Shandong, China). Antibodies against phospho-IκBα, phospho-NF-κB p65 and phospho-IKKβ were obtained from Cell Signaling Technology (Beverly, MA, USA). Blood Urea Nitrogen (BUN) and Serum Creatinine Determination (SCr) assay kit reagents were supplied by were purchased from the Institute of Jiancheng Bioengineering (Nanjing, China). DMEM medium and fetal bovine serum (FBS) were products of Gibco Corporation (USA). Zymosan A, nitroblue tetrazolium (NBT) and the other reagents were all purchased from Sigma-Aldrich Chemical Co. (USA). All other reagents were of analytical grade.

### Animals

Eight-week-old BALB/c mice were purchased from Vital River Laboratory Animal Technology Co. Ltd. (Certificate No. 0247652). All animals were acclimated for at least 1 week at a temperature of 24 ± 1 °C and humidity of 55 ± 5%. The animals were maintained with free access to standard diet and tap water.

### Ethics statement

All the animal experiments in our study were performed in accordance with the Guide for the Care and Use of Laboratory Animals, formulated by the National Institutes of Health, USA, and approved by the Office of Experimental Animal Management Committee of Shandong Province, China (certificate No. SYXK (Lu) 20090015) and local Animal Ethical Committee.

## Experimental design

### *In vivo* study

#### Model of LPS-induced acute kidney injury

The mice were intragastrically (i.g.) given 20, 40 and 80 mg/kg rhein, which was dissolved in 5% carboxymethylcellulose sodium (CMCS) as vehicle. The rhein doses adopted here was based on the preliminary experiments in this laboratory. Rhein and the vehicle were given once a day at 9 a.m. by oral gavage for 7 days. After the last of administration, all mice except the control group received a single intraperitoneal injection of 10 mg/kg of LPS. The mice in control group were given an intraperitoneal injection of saline. Twelve hours after the LPS injection, blood samples were collected from the retroorbital venous plexus and centrifuged at 4 °C for 10 min at 1400 × g to prepare serum, the serum was stored at −80 °C in polystyrene tubes and the kidneys were quickly removed, frozen in liquid nitrogen and stored at −80 °C for later biochemical analysis.

#### Model of polymicrobial sepsis caused by cecal ligation and puncture

The CLP procedure followed the original report by Baker *et al.*^22^, and modified by Ondiveeran^23^. Mice are anesthetized with ketamine (75 mg/kg, intramuscular, i.m.) and xylazine (10 mg/kg, i.m.), and placed in supine position, with their feet taped to ensure a stable position. Clean the abdomen and make a 15 mm midline incision to expose and isolate the cecum. Ligate the cecum with a 4-0 silk suture at 5.0 mm from the cecal tip, and then puncture the ligated cecal stump once with a 22-gauge needle. Put the cecum back immediately to its normal intra-abdominal position. Sham-operated mice underwent opening of the peritoneum and bowel exposure, but without ligation and puncture. Close the incision site and resuscitate mouse with 0.5 ml sterile normal saline solution for fluid resuscitation after surgery. Return the mouse back to a clean cage with free access to food and water.

Mice were randomly divided into six groups (n = 10 mice/group): In sham group, mice underwent a sham operation, receiving 5% CMCS as control vehicle. In sham plus rhein group, mice underwent a sham operation and intragastrically (i.g.) given rhein (80 mg/kg) dissolved in 5% CMCS. In CLP group, mice were exposed to CLP, receiving control vehicle. In CLP plus rhein (20 mg/kg) group, in CLP plus rhein (40 mg/kg) group and in CLP plus rhein (80 mg/kg), mice were performed by CLP surgery, and orally administered rhein 20 mg/kg, 40 mg/kg and 80 mg/kg respectively, immediately after operation. 12 hours after CLP, no mortality in each group, kidney tissue and blood samples were collected for quantification of biochemical analysis.

The cells present in the peritoneal cavity of each group were collected by introducing Hanks balanced salt solution. Peritoneal macrophages were isolated and the phagocytic activity was measured using the assay system described by Shin^24^. Cells were incubated with 5 × 10^6^ particles of opsonized zymosan and 0.5 mg/ml of NBT. After 1 h incubation, plates were centrifuged at 4 °C to stop the ingestion of zymosan and supernatant was removed by flipping. NBT reduction was assayed colorimetrically, in brief, intracellular blue-black formazan deposits were solubilized with 140 μl of 2 M sodium hydroxide (NaOH) and 120 μl of dimethylsulfoxide (DMSO), and the absorption of the formazan solution was measured at 630 nm.

### Histopathological examination

For histopathological observation, the mice were sacrificed and kidneys were removed 12 hours after LPS challenge. Each tissue was fixed in 10% formalin, embedded in paraffin, sectioned and then stained with hematoxylin and eosin for morphological examination. The semiquantitative score for kidney injury was calculated for each animal by a blinded observer. The percentages of tubules that displayed cellular necrosis, loss of brush border, interstitial edema, vacuolization, and tubule dilatation were scored as follows: 0 = none, 1 = 0–20%, 2 = 20%–50%, 3 = 50%–70%, 4 = more than 70%. For each animal, at least 10 fields were examined.

### Biochemical measurements

Renal function injury and inflammatory cytokine were assessed in mice subjected to LPS or CLP at the end of experiment. BUN and SCr, as important index of renal injury severity, were used for the assessment of renal function. The concentration of BUN and SCr in serum was measured with SpectraMax M2 Multi-Mode Micro plate Reader and their concentrations were expressed as mmol/L and μmol/L respectively.

Renal tissues were rapidly excised and homogenized in phosphate-buffered saline (pH 7.4, w/v; 1 g tissue with 9 ml PBS) with an Ultra-Turrax T25 Homogenizer. After centrifugation at 10,000 g for 10 min at 4°C, the supernatant was used to determine the concentration of TNF-α and IL-1β, using ELISA kits according to the manufacturer’s instructions.

### Determination of white blood cell counts

At the completion of the CLP experiments, blood was harvested by cardiac puncture with a heparinized syringe. The white blood cell count (WBC; neutrophils, monocytes, lymphocytes) was performed using an automated cell counter adjusted for murine blood cells (PE-6800VET Animal Blood Counter, San Feng Company, China).

### Immunohistochemical studies

Kidneys collected from the mice in LPS-induced acute kidney injury experiment were subjected to immunohistochemical staining. The slides were incubated with primary antibodies, phosphorylation of NF-κBp65 diluted to 1:200 in PBS for 16 h at 4°C, then washed three times with PBS, and incubated for 30 min with biotin-conjugated secondary antibodies for 1 h. The slides were washed again with PBS. Finally, the samples were developed by using diaminobenzidine (DAB). Hematoxylin was used as the counter stain.

### Western blot analysis

For western blot analysis of collected kidneys tissues after LPS challenge, the tissues were washed in PBS, added to a homogenizer, mixed with pre-cooled lysis buffer and then homogenized on ice followed by centrifugation at 12000 × g for 30 s. Equal amounts of protein were separated by SDS-PAGE and analyzed by Western blot using specific antibodies to phosphorylated IκBα, IKKβ and NF-κBp65. Horseradish peroxidase-conjugated anti-rabbit IgG antibody (Santa Cruz Biotechnology) was used as a secondary antibody. Protein was detected using an enhanced chemiluminescence detection kit (Beyotime Institute of Biotechnology) and were scanned and quantified with DigDoc100 program. Data were normalized against those of the corresponding β-actin bands. Results were expressed as fold increase over control group.

### *In vitro* study

#### Cell culture and treatment

Human renal proximal tubular epithelial cells (HK-2 cells), were purchased from ScienCell Research Laboratories, USA. HK-2 cells were cultured in DMEM medium supplemented with 10% heat-inactivated FBS and 1.0% penicillin-streptomycin solution in a humidified incubator with 5.0% CO_2_ at 37 °C. Cells from passages 3 to 5 after recovery were used throughout study.

#### MTT assay for cell viability

The 3-(4, 5-dimethyl-2-thiazolyl)-2, 5-diphenyl tetrazolium bromide (MTT) assay was used to measure cell proliferationindicate. Cells were seeded at 10^4^ cells/well in 96-well plates with serum-free medium for 24 h incubation. Cells were incubated in presence or absence of different concentrations of rhein(10, 20 and 40 μM) for 24 h, then incubated with or without 1 μg/ml LPS for another 24 h. Then 20 μl of MTT (5 mg/ml) was added to each well and incubation continued at 37 °C for additional 4 h. After removing the supernatant, 100 μl of DMSO was added to dissolve the reduced formazan. The absorbance at 570 nm wavelength was measured by using a microplate reader. The control group consisted of untreated cells was considered as 100% of viable cells. Results are expressed as percentage of viable cells when compared with control groups.

#### Cytokine assays *in vitro*

HK-2 cells were seeded in a 96-well plate at the density of 5 × 10^5^ cells/ml. After 1 h incubation, cells were treated with LPS (1 μg/ml) and rhein (10–40 μM) for 24 h. 100 μl of supernatant were taken out. The levels of MCP-1 and IL-8 in the supernatant were determined using commercial enzyme-linked immunosorbent assay (ELISA) kits according to the manufacturer’s instructions.

#### Western blot analysis

After indicated treatment, cells treated with different concentrations of rhein (10, 20 and 40 μM) followed by LPS treatment (1 μg/ml), were lysed and homogenized in lysis buffer immediately. Cells proteins were extracted according to the instruction of the Total Protein Extraction Kit. The BCA protein assay kit was used todetermine the protein concentration. Cell lysates contain equal amount of proteins were separated by SDS-PAGE gel electrophoresis and electro-blotted onto a nitrocellulose membrane. The protein levels of phosphorylated IκBα, IKKβ and NF-κB p65 were detected by Western blot analysis.The concentrations were calculated from the standard curves. Standard procedures were used for the Western blotting as described above.

#### Statistical analysis

All data are expressed as means ± SD. Statistical significance of differences between groups was determined by ANOVA. Differences were considered significant at *p* < 0.05.

## Result

### Effects of Rhein on LPS-Induced Renal Injury

**Figure 2:**
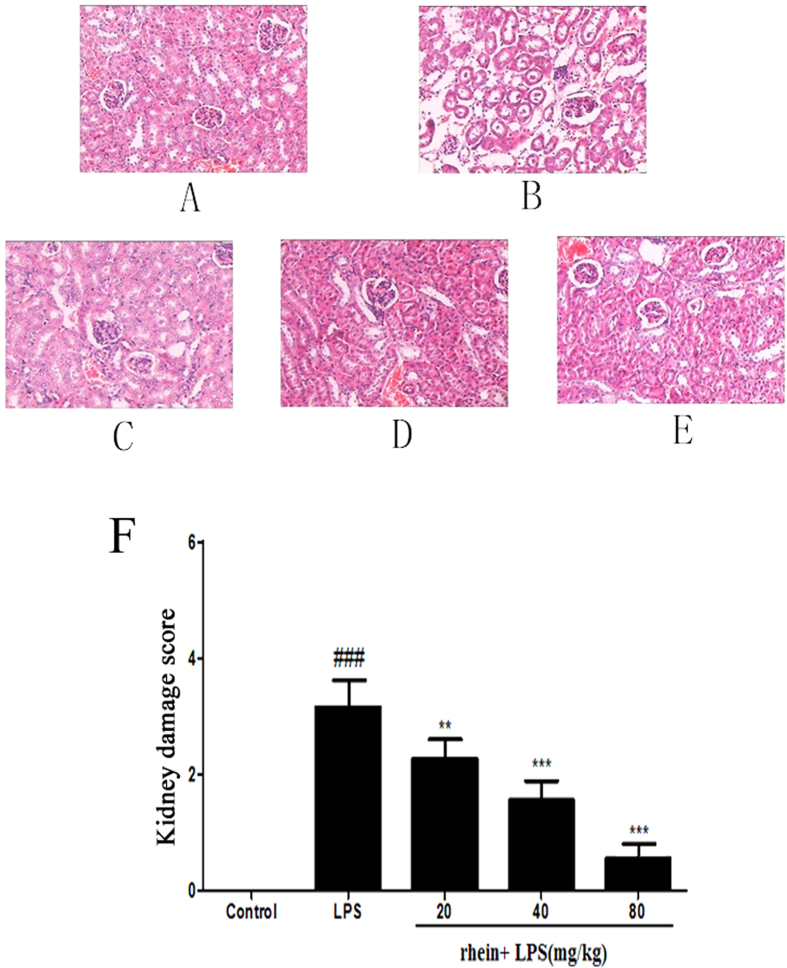
Effect of rhein on kidney injury after LPS administration. Representative histological changes in kidneys obtained from mice of different groups (**A**) Control group; (**B**) LPS group; (**C**) Rhein (20 mg/kg) + LPS group; (**D**) Rhein (40 mg/kg) + LPS group; (**E**) Rhein (80 mg/kg) + LPS group.The sections shown were harvested 12 h after LPS injection and stained with H&E. Magnification: ×400. (**F**)Pathological score of representative kidney samples of each group. Data are represented as mean ± SD of 5 animals of each group. ###*P* < 0.001 versus control group; **P < 0.01 and ***P < 0.001 versus LPS group.

Histopathological change is a direct indication of renal injury. The H&E-stained renal tissues appeared to have normal kidney tubules in the control group samples (Fig. 2A). In contrast, it was demonstrated that LPS-induced histopathological changes in the renal tissues, such as the edema of renal tubular epithelial cells, glomerular atrophy, dilation of renal capsule cavity, destruction of tubular structures, the epithelial cells of the local focal necrosis collapse, renal interstitial edema of epithelial cells (Fig. 2B). However, pretreatment with rhein (20, 40 and 80 mg/kg mg/kg) significantly diminished LPS-induced epithelial atrophy and necrosis and interstitial edema to varying degrees (Fig. 2C–E). There was a significantly reduced in the score of kidney pathological damage (Fig. 2F).

### Effects of Rhein on LPS-Induced Renal Dysfunction

**Figure 3:**
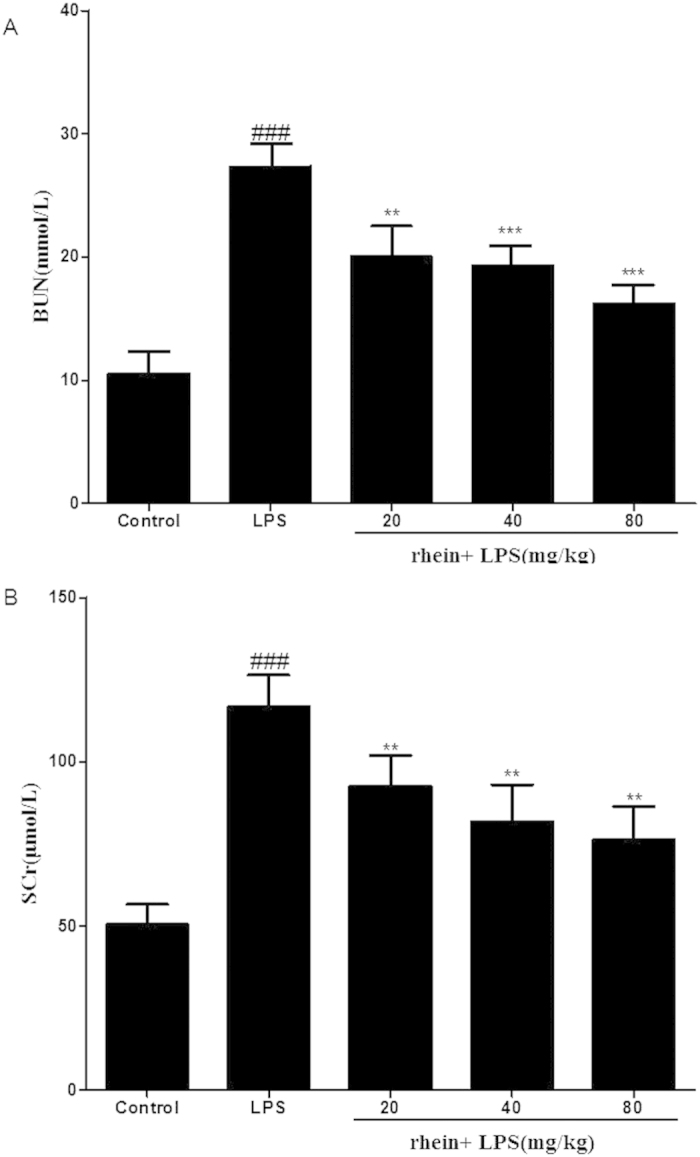
Effects of rhein on serum BUN (**A**) and SCr(**B**). Data are represented as mean ± SD of 10 animals of each group. ###*p* < 0.001 compared to control group; **p < 0.01 and ***p < 0.001 compared to LPS group.

BUN and SCr levels were used for the assessment of renal function. The BUN and SCr levels in the LPS-induced group were found to be significantly higher than the control groups; however treatment with rhein caused significant dose-dependent reduction in both BUN and SCr levels (Fig. 3).

### Effects of Rhein on LPS-Induced Pro-inflammatory Cytokines

**Figure 4:**
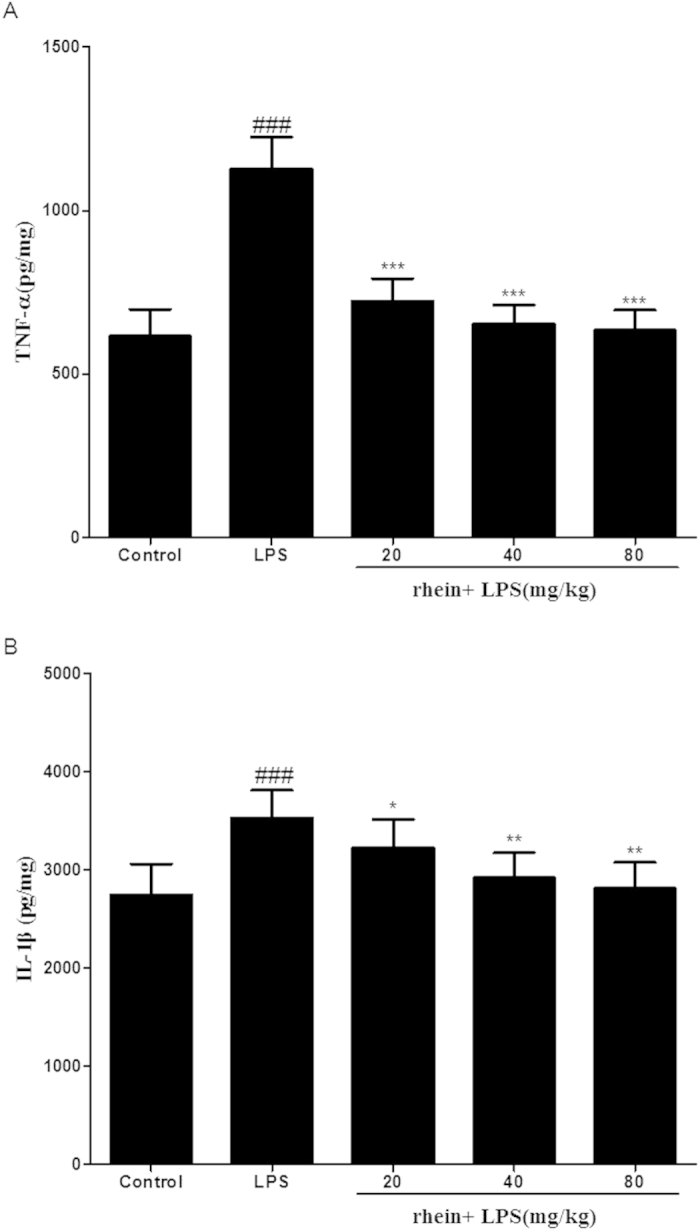
The effect of rhein on TNF-α (**A**) and IL-1β (**B**) levels after LPS challenge. Quantitation of TNF-α and IL-1β in renal tissue was performed by ELISA. Data are represented as mean ± SD of 10 animals of each group. ###*p* < 0.001 compared to control group; *p < 0.05, **p < 0.01 and ***p < 0.001 compared to LPS group.

To investigate the pro-inflammatory molecules generated by LPS renal injury, the cytokine levels of TNF-α and IL-1β in renal tissue were measured. LPS-treated mice had significantly increased levels of TNF-α and IL-1β at 12 h after lipopolysaccharide injection, while rhein pretreatment significantly reduced TNF-α and IL-1β production by lipopolysaccharide treatment in a dose-dependent manner (Fig. 4).

### Effect of Rhein on NF-κB signal pathway in LPS-Induced AKI

**Figure 5:**
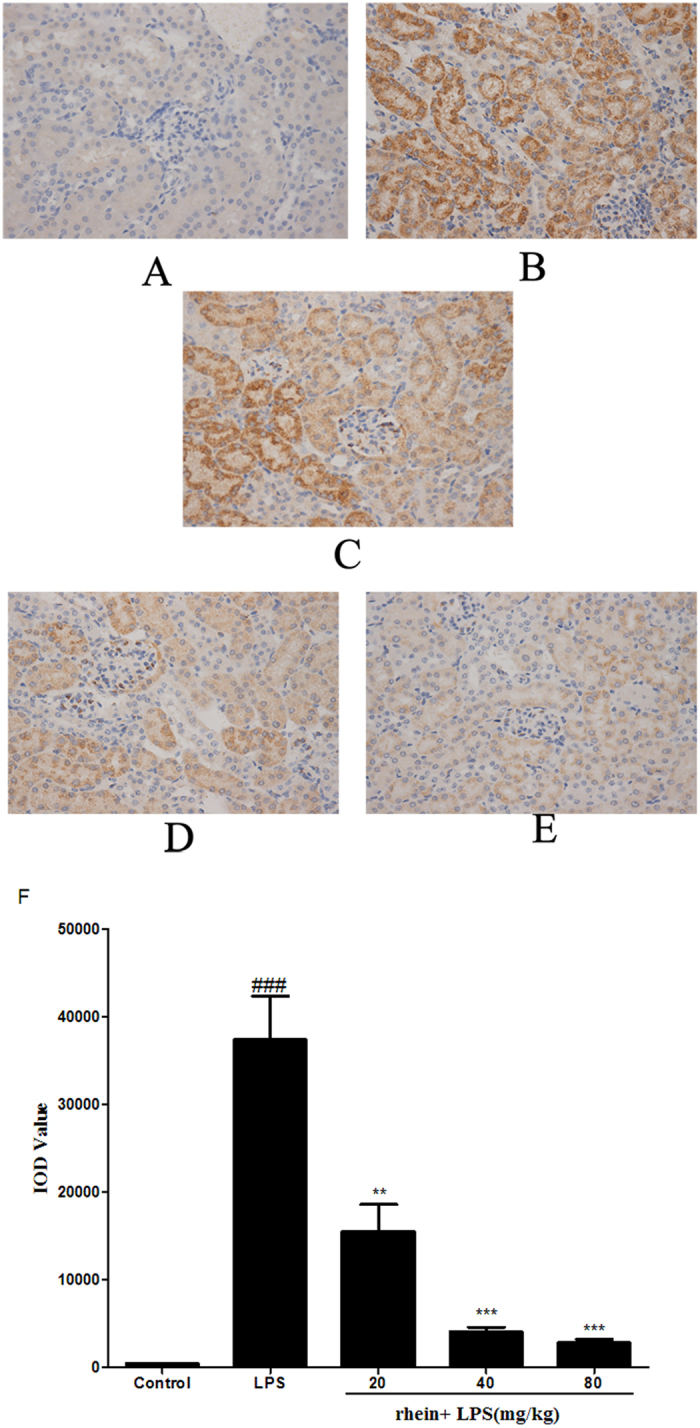
Effect of rhein on phospho-NF-κB p65 localization and expression in AKI by immunohistochemistry (magnification × 400). (**A**) Control group; (**B**) LPS group; (**C**) Rhein (20 mg/kg) + LPS group; (**D**)Rhein (40 mg/kg) + LPS group. (**E**) Rhein (80 mg/kg) + LPS group. (**F**) IOD values of phospho-NF-κB p65 staining. Data are represented as mean ± SD of 5 animals of each group, ### *P* < 0.001 versus control group; **p < 0.01 and ***P < 0.001 versus LPS group.

To gain a better insight into the potential mechanisms underlying the observed beneficial effects of rhein on the sepsis-associated AKI, we investigated the effects of this compound on NF-κB signaling pathways in the kidneys of mice. Immunostaining for phosphorylated NF-κB p65 demonstrated its expression and localization in kidney sections. Staining for phosphorylated NF-κB p65 in nuclei and cytoplasm of proximal convoluted tubule and renal glomerulus was more pronounced in LPS-induced group mice than in control mice. As shown in Fig. 5, rhein administration attenuated the phosphorylated NF-κB p65 staining.

**Figure 6:**
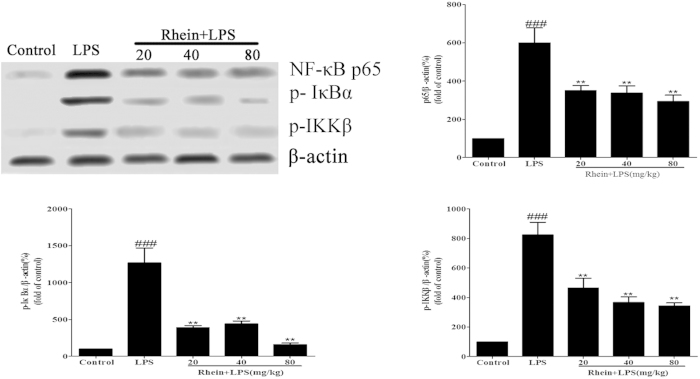
Effects of rhein on the expression of NF-κB p65, p-IκBα and phospho-IKKβ in LPS-induced AKI. Protein extracts were obtained from kidney tissues and proteins expression level was detected by Western blotting analysis. All data represent the means ± SD from three separate experiments. ##*p* < 0.01 and ###*p* < 0.001 compared to control group; **p < 0.01 compared to LPS group.

NF-κB signal pathway relevant protein expression in AKI mice kidney tissue was examined by using western blot analysis. As shown in Fig. 6, The protein expression of phospho-IKKβ, phospho-IκBα and phospho-NF-κB p65 were increased in LPS-induced group mice compared to control group treated with saline. This indicates that NF-κB activity was increased. Administration of rhein down-regulated the expression of phosphorylated NF-κB p65, IKKβ and IκBα. These results suggest that rhein had a protective effect on mice with LPS-induced AKI through suppression of NF-κB signal pathway.

### Effect of Rhein on CLP-induced septic-AKI

**Figure 7:**
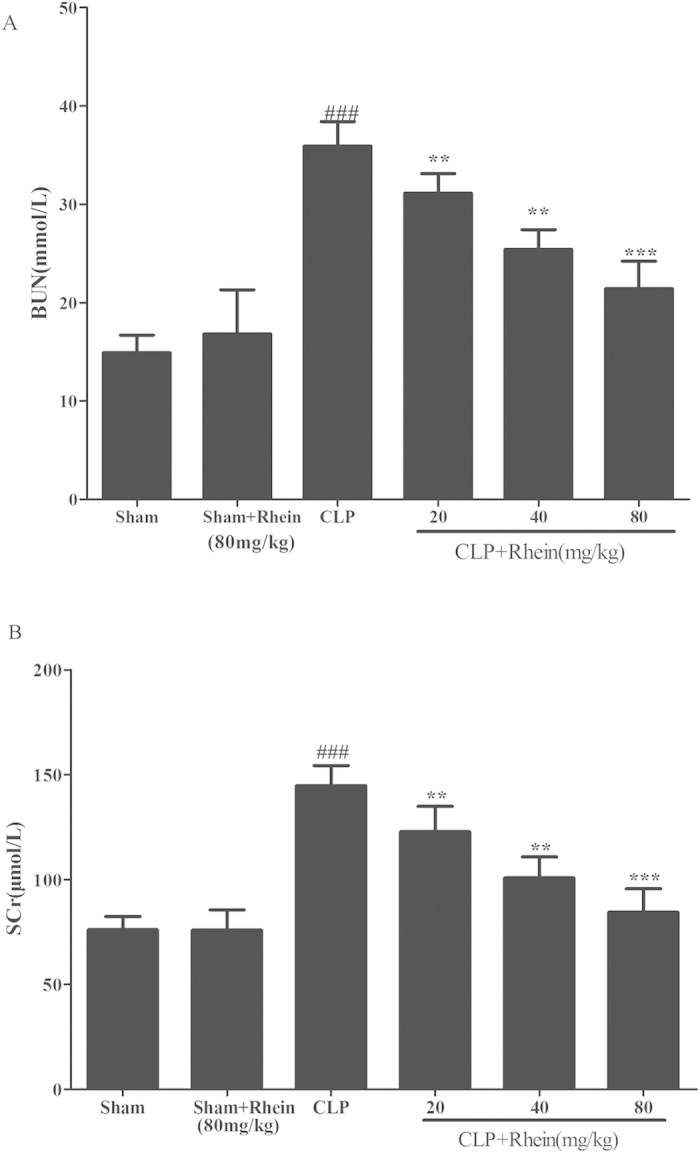
Effects of rhein on serum BUN (**A**) and SCr(**B**) in CLP model. Data are represented as mean ± SD of 10 animals of each group. ###*p* < 0.001 compared to sham group; **p < 0.01, ***p < 0.001 compared to CLP group.

To further investigate the effect of rhein on AKI induced by polymicrobial sepsis, we established murine CLP model. BUN and SCr were determined to analyze kidney function. As shown in Fig. 7, CLP induced significant increases in both BUN and SCr levels compared with sham group. The increases were significantly attenuated by treatment with rhein. While only rhein treatment has no significant effect on BUN and SCr levels, these results indicate that rhein has a protective effect on CLP-induced AKI without nephrotoxicity at high doses.

**Figure 8:**
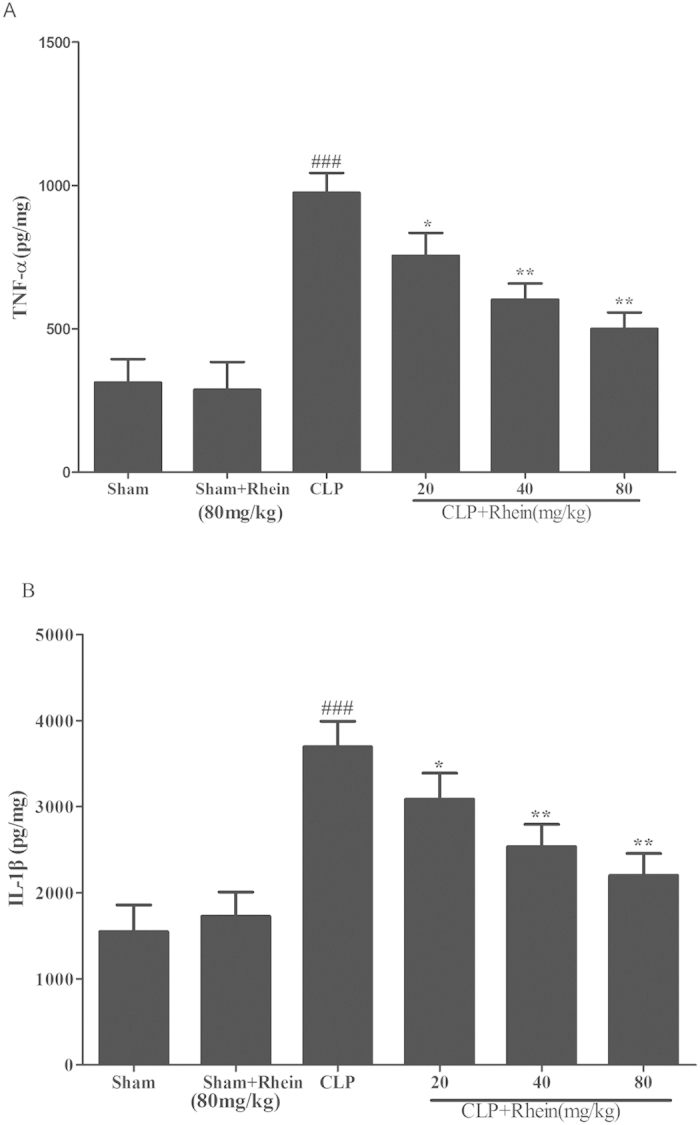
Effects of rhein on TNF-α (**A**) and IL-1β (**B**) levels after CLP challenge. Quantitation of TNF-α and IL-1β in renal tissue was performed by ELISA. Data are represented as mean ± SD of 10 animals of each group. ###*p* < 0.001 compared to sham group; *p < 0.05, **p < 0.01 and ***p < 0.001 compared to CLP group.

**Figure 9:**
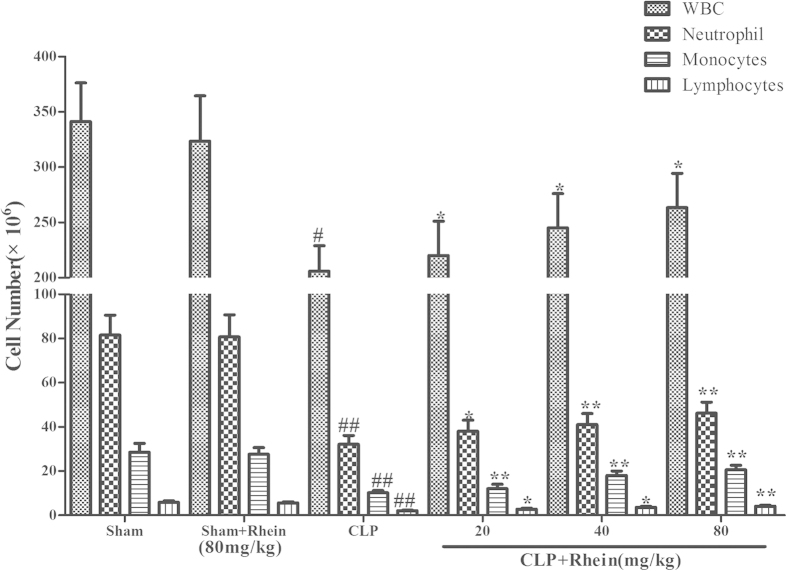
The effect of rhein on peripheral white blood cell counts in CLP-induced sepsis. Blood samples were withdrawn by cardiac puncture with a heparinized syringe at 12 h after the CLP surgery. total and differential cell counts were measured. Data are represented as mean ± SD of 10 animals of each group. #*p* < 0.05 and ##*p* < 0.01 compared to sham group; *p < 0.05, **p < 0.01 and ***p < 0.001 compared to CLP group.

The increase of pro-inflammatory cytokines and reduction of circulatory white blood cell (WBC) count are typical signs of pro-inflammatory response and onset of sepsis. In our study, pro-inflammatory cytokines (TNF-α and IL-1β) and WBC count were detected for the evaluation of the severity of sepsis in each group. The results showed that the levels of TNF-α and IL-1β in kidney were low and comparable in sham mice treated with either vehicle or rhein. By contrast, a dramatic increase levels of TNF-α and IL-1β were observed in CLP group 12 h after the surgery. The elevated levels of TNF-α and IL-1β were significantly inhibited by treatment with rhein (Fig. 8). As shown in Fig. 9, peripheral WBC count at 12 h after CLP surgery was significantly decreased. Rhein significantly increased total leukocyte number ,and the following differential count showed that both monocytes and neutrophil counts were significantly increased after rhein treatment, while rhein treatment only in sham mice did not alter the WBC level.

**Figure 10:**
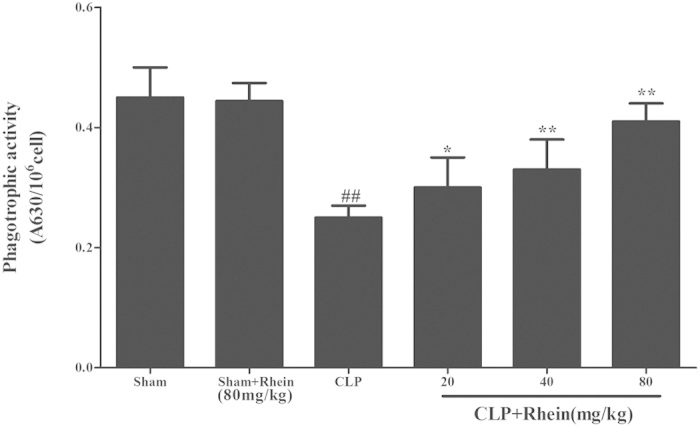
The effect of rhein on peritoneal macrophage phagocytic activity in CLP-induced mice sepsis. Macrophages harvested 12 h after CLP were incubated with zymosan and NBT. Phagocytosis was measured as OD 630 nm. Data are expressed as mean ± SD (n = 10). ##*p* < 0.01 compared to sham group; *p < 0.05,**p < 0.01 and ***p < 0.001 compared to CLP group.

Macrophages play a critical role in regulating the inflammatory response subsequent to an insult. The phagocytosis was assayed to further study the effect of rhein on phagocytic activity of peritoneal macrophages in CLP-induced sepsis. The results from the phagocytosis experiments (Fig. 10) suggested that macrophage phagocytic function was suppress in CLP mice. Treatment with rhein effectively enhanced phagocytic function of peritoneal macrophages. Above results demonstrated that rhein could effectively attenuate the severity of inflammatory responses induced by CLP surgery.

### Effects of Rhein on cells viability

**Figure 11:**
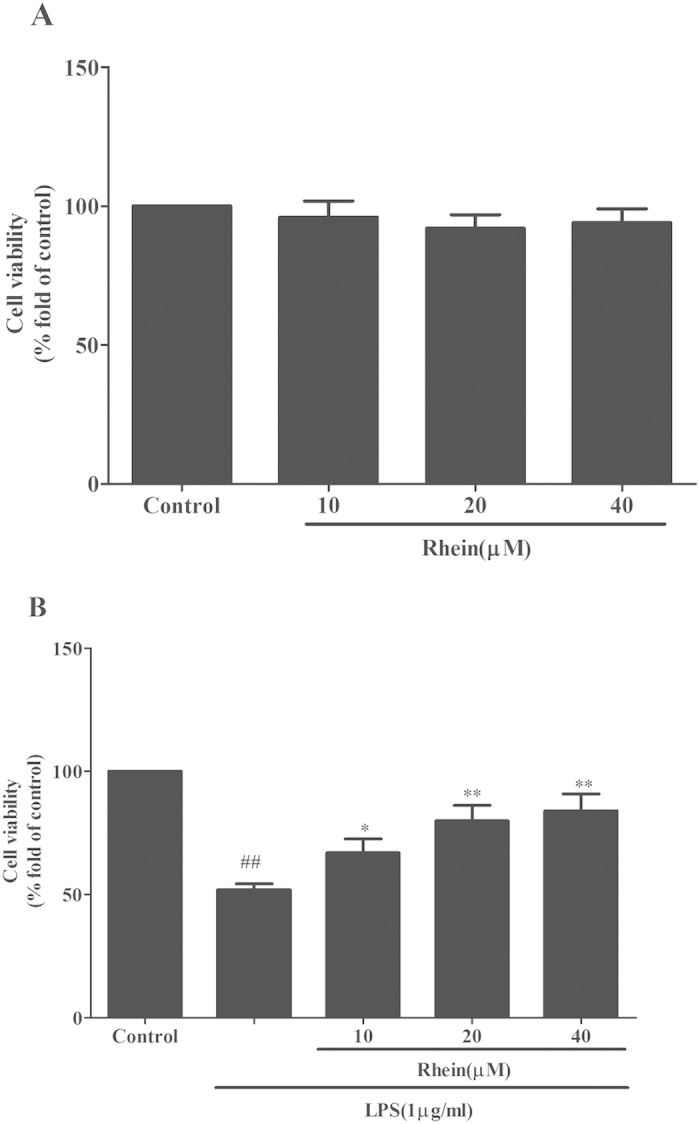
The effect of rhein on HK-2 cells viability were tested by MTT assay. (**A**)Effect of rhein on HK-2 cells proliferation in normal condition by MTT assay.(**B**) Effect of rhein on LPS-induced HK-2 cells proliferation by MTT assay. Results are expressed as percentage of viable cells when compared with control groups. Data are expressed as mean ± SD*. ##p* < 0.01 vs. control group, *p < 0.05 and **p < 0.01 vs. LPS alone.

HK-2 cells proliferation was evaluated using MTT assay. The proliferation of HK-2 cells cultured under normal condition was not altered by rhein treatment (Fig. 11A). It is revealed that rhein did not exhibit cytotoxicity against HK-2 cells. In contrast, compared with control, LPS could markedly inhibit HK-2 cells proliferation, which was significantly enhanced by rhein in a concentration dependent manner (Fig. 11B).

### Effect of Rhein on cytokines release in LPS-induced HK-2 cells

**Figure 12:**
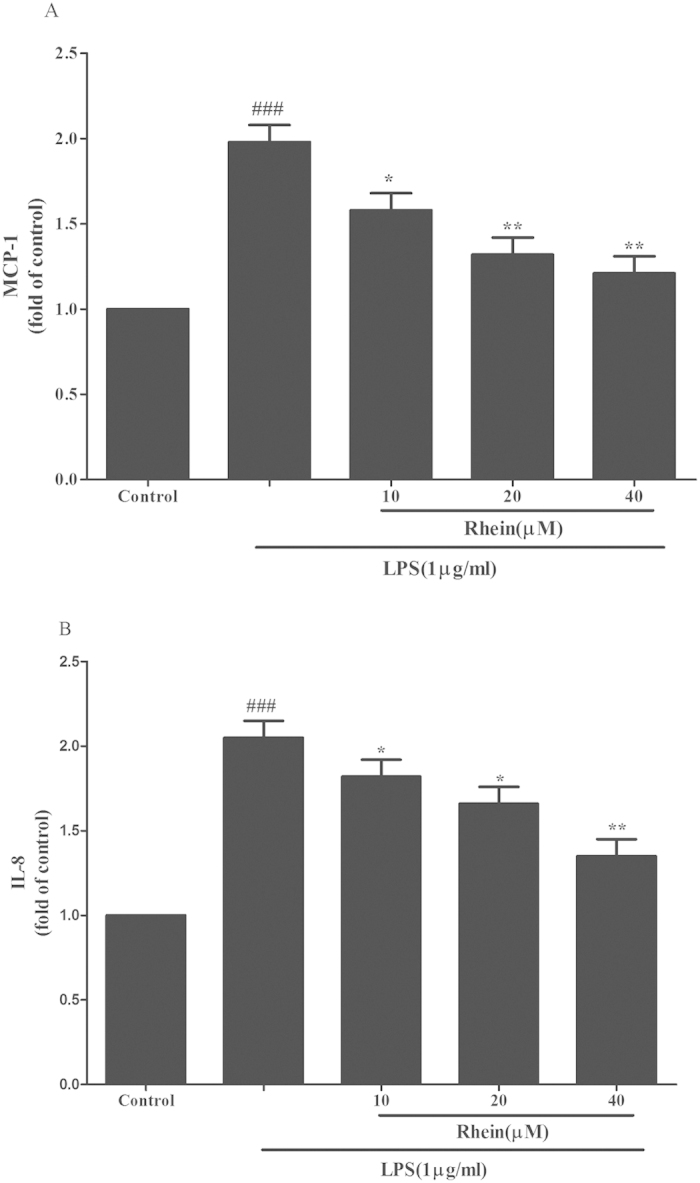
The effect of rhein on MCP-1 (**A**) and IL-8 (**B**) release induced by LPS in HK-2 cells. Cells were treated with LPS with or without rhein (10, 20, and 40 μM) for 24 h. 100 μl of culture medium in each group was taken out to measure the levels of MCP-1 and IL-8 using ELISA kits. Data are represented as mean ± SD of three independent experiments. *###p* < 0.001 vs. control group, *p < 0.05, **p < 0.01 vs. LPS alone.

The levels of MCP-1 and IL-8 were measured by ELISA after 24 h treated with LPS with or without rhein. As shown in Fig. 12, rhein suppressed the release of MCP-1 and IL-8 in LPS-stimulated HK-2 cells in a concentration-dependent manner.

### Effect of Rhein on NF-κB signal pathway in LPS-Induced HK-2 cells

**Figure 13:**
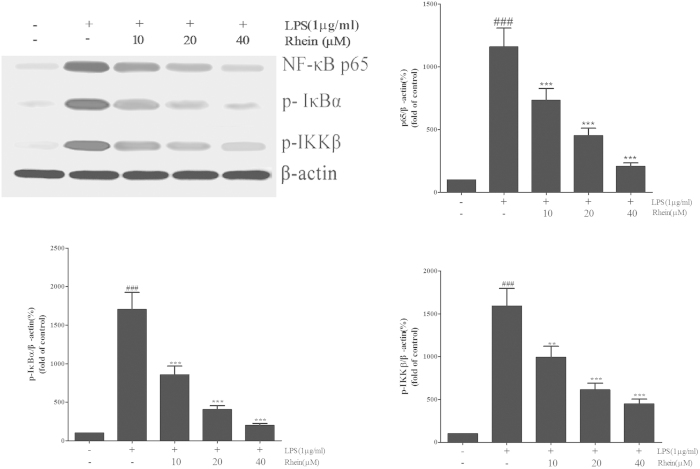
The effect of rhein on NF-κB signal pathway in LPS-induced HK-2 cells. The expression of NF-κB p65, p-IκBα and phospho-IKKβ were assessed by Western blot analysis. β-actin was used as an internal control. Results are expressed as fold increase over control group. Data are represented as mean ± SD of three independent experiments. ##p < 0.01 vs. control group, **p < 0.01 vs. LPS alone.

To verify whether the actions of rhein attribute to the inhibition of NF-κB signal pathway, the key protein molecules were detected by western blot. As shown in Fig. 13, rhein significantly suppressed the expression of phosphorylated NF-κB p65, IKKβ and IκBα induced by LPS in a dose-dependent manner.

## Discussion

Sepsis has been identified as the most common cause of AKI in intensive care units. Moreover, the combination of sepsis and AKI is associated with a very high mortality rate^25^. Considering the high incidence and related morbidity and mortality of sepsis associated AKI, there is an urgent medical need to investigate novel pharmacological interventions to treat or prevent AKI. The current study was designed to elucidate the role of rhein in two different mouse models of experimental sepsis and *in vitro* HK-2 cells.

We first developed a model of severe LPS induced AKI in young mice. LPS, an endotoxin, is a major component of the outer membrane of Gram-negative bacteria, which are considered the main aetiology of sepsis.The mechanism of LPS-induced kidney injury could be that LPS can stimulate the synthesis and release of pro-inflammatory cytokines by tissue macrophages and circulating monocytes and lead to a transient immune activation, which is characterized by elevated levels of circulating TNF-α, IL-1β and IL-6^26^. The excessive release of these inflammatory mediators can result in a lethal systemic inflammatory response syndrome, which leads to shock, vascular dysfunction, disseminated intravascular coagulation and multiple organ dysfunction/injury^27^. Therefore, experimental endotoxemia induced kidney injury by LPS is an ideal model which is widely used to study the mechanisms of septic AKI^28^,^29^. It is reported that the biological activity of LPS and the sensitivity to endotoxin varies significantly between species and strains^30^,^31^.

In the present study, we successfully established murine AKI model by treating BALB/c mice with 10 mg/kg LPS (from Escherichia coli 055:B5) according to preliminary work and the description in previous literature^32^,^33^. Histopathology examination showed that the glomerular structure is destroyed, renal tubular epithelial cell degenerated and there were severe intracellular edem and congestion within renal tubule and renal interstitium in LPS-induced group. Treatment with rhein, however, lightened the severity of the lesions, reduced the extent of renal injury and alleviated the infiltration of inflammatory cells. In addition the levels of BUN and SCr as an index of renal injury were measured. From the experimental data, The BUN and SCr levels in the LPS-induced group were found to be significantly higher than the control groups; however treatment with rhein caused statistically significant reduction in BUN and SCr levels. All these confirmed that we had established a suitable animal model of LPS-induced AKI and rhein had beneficial effect on LPS-induced AKI.

Although sepsis can occur in previously healthy and/or young individuals, the elderly, surgical patients and patients with chronic diseases are predisposed to suffer from this condition^34^,^35^. Therefore, we wished to strengthen our study by confirming our observations in a clinically relevant model of severe polymicrobial sepsis caused by CLP. In this study, we found that rhein could also reduce the increase of BUN and SCr induced by CLP, attenuate leukocyte infiltration and stimulate the phagocytic function of peritoneal macrophages. It is further confirmed that rhein exert protective effect on septic AKI due to its anti-inflammatory and immunomodulatory properties.

The pathophysiology of AKI in sepsis is complex and multi-factorial, growing evidence has shown that pro-inflammatory cytokines have a prominent role in sepsis-induced AKI^36^. Host responses to infectious challenge result in the excessive release of inflammatory mediators, such as TNF-α and IL-1β, which triggers the pathophysiological abnormities of sepsis. Elevated levels of these cytokines play a key role for the pathogenesis of AKI and other systemic dysfunctions in sepsis^37^. Therefore, the inhibition of inflammatory mediators could be considered an effective treatment for septic AKI. Our results showed that rhein treatment decreased the level of TNF-α and IL-1β in the two sepsis models *in vivo*, which suggest that the protection of rhein against septic-AKI are related to down regulation of TNF-α and IL-1β level.

TNF-α and IL-1β are major mediators responsible for the expression of chemokines such as MCP-1 and IL-8, which are important chemoattractant for neutrophils, monocytes/macrophages and T lymphocytes during renal inflammation. These chemokines could initiate local infiltration of monocytes/macrophages after its activation. In HK-2 cells, rhein could attenuate LPS-induced the release of MCP-1 and IL-8 to inhibit the infiltration of monocytes/macrophages. Our data are consistent with the result obtained from *in vivo* studies.

It is well known that genes encoding of many pro-inflammatory cytokines, such as TNF-α, IL-1β and MCP-1, are all to be under the control of NF-κB transcription factors, which is one of the most important and widely used transcription factors and is involved in the regulation of gene expression in LPS-induced inflammation response during kidney injury and pathophysiology of sepsis^38^,^39^. TNF-α interacts physically with NF-κB signaling pathway. The TNF-α can activate NF-κB signaling pathway which in turn can promote the expression of the other inflammatory cytokines and enlarges the inflammatory response in AKI^40^,^41^. In order to determine whether rhein affect NF-κB activity, we planned to investigate the key molecules that regulate NF-κB activation.

In this study, we observated the activation of NF-κB signal in kidney tissue of sepsis AKI mice and cultured HK-2 cells by relative protien expression and p65 translocation measurement in the presence or absence of treatment of rhein. We observed that the phosphorylation of p65, IκBα, and IKKβ were stimulated by LPS both *in vivo* and *in vitro*. LPS induced an increased expression of on phosphorylation IKKβ and IκB as well as an increased nuclear translocation of the phosphorylated NF-κB subunit p65. IKKs can phosphorylate the inhibitory IκBα protein and this phosphorylation results in the dissociation of IκBα from NF-κB, which liberate NF-κB to the nucleus and activate NF-κB target inflammatory ngenes. Our finding suggested that the effect of rhein on preventing inflammatory response in septic AKI may due to inhibit the NF-κB signal pathway activation by regulating phosphorylation of IKKβ, IκBα and p65 or block NF-κB p65 translocation.

In conclusion, we have proved that treatment of rhein attenuates sepsis-induced acute kidney injury, so that it has a significant effect on development and progression of septic AKI. The underlying mechanism of rhein on sepsis-induced kidney injury may be closely related with its anti-inflammatory roles. Rhein could inhibit the production of pro-inflammatry cytokines and attenuate leukocyte infiltration by decreasing the activation of NF-κB through restraining the expression and phosphorylation of the relevant proteins in NF-κB signal pathway, hindering transcription of NF-κB p65. This evidence suggests that rhein has a potential application for reducing the inflammatory processes to treat endotoxemia-associated acute kidney injury.

## Additional Information

**How to cite this article**: Yu, C. *et al.* Rhein prevents endotoxin-induced acute kidney injury by inhibiting NF-κB activities. *Sci. Rep.* **5**, 11822; doi: 10.1038/srep11822 (2015).

## Figures and Tables

**Figure 1:**
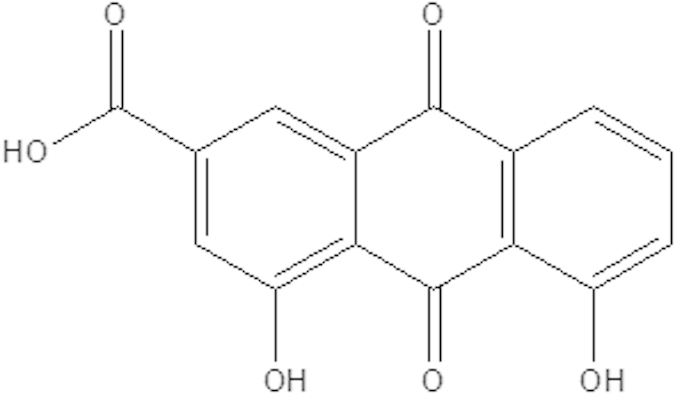
The chemical structure of rhein. (C_15_H_8_O_6_, molecular weight = 284.22).
